# Relationship between Parental Concerns about Social–Emotional Reciprocity Deficits and Their Children’s Final ASD Diagnosis

**DOI:** 10.3390/children10111786

**Published:** 2023-11-06

**Authors:** Ronit Saban-Bezalel, Einat Avni, Esther Ben-Itzchak, Ditza A. Zachor

**Affiliations:** 1 Bruckner Center for Autism Research, Department of Communication Disorders, Ariel University, Ariel 40700, Israel; ronitsa@ariel.ac.il; 2 The Autism Center/ALUT, Department of Pediatrics, Shamir (Assaf Harofeh) Medical Center, Zerifin 70300, Israel; einata@shamir.gov.il (E.A.); dzachor@shamir.gon.il (D.A.Z.); 3 The Sackler Faculty of Medicine, Tel Aviv University, Tel Aviv 6997801, Israel

**Keywords:** parental concerns, social–emotional reciprocity, ASD, non-ASD, early and middle childhood

## Abstract

Concerns raised by parents regarding their child’s development are compatible with the child’s final diagnosis of autism spectrum disorder. A better understanding of the relationship between parental concerns and a final diagnosis of autism spectrum disorder is therefore critical. In the current study, we compared the frequencies of parental concerns related to DSM-5 criteria for autism spectrum disorder between pair-matched groups with and without a final diagnosis of autism spectrum disorder and determined which parental concerns predicted a final diagnosis of autism spectrum disorder. The cohort included 80 participants (48–154 months of age, IQ > 70) assessed for a possible autism spectrum disorder diagnosis. Parental concerns were retrieved from the free-description portion of the introductory questions of the Autism Diagnostic Interview-Revised and analyzed to assess whether they corresponded to any of the seven DSM-5 criteria for ASD. The two groups only differed in the frequency of parental concerns relating to deficits in social–emotional reciprocity. Parents of children diagnosed with autism spectrum disorder were four times as likely to report deficits in social–emotional reciprocity. This finding highlights the significance of parental concerns regarding deficits in social–emotional reciprocity in predicting a final diagnosis of autism spectrum disorder.

## 1. Introduction

Autism spectrum disorder (ASD) reflects a continuum of impaired social communication skills (SCIs) and restricted/repetitive behaviors (RRBs) [1]. ASD is a heterogeneous condition with a spectrum of severities [2]. It is well-known that early diagnosis of ASD is crucial, as it allows for prompt enrollment in intervention programs, leading to early intervention, which results in better outcomes [3,4,5]. A comprehensive autism diagnosis procedure requires using gold-standard tools and integrating information from multiple sources, with parents playing an essential role in this process. Despite the widespread use of a comprehensive diagnosis procedure for autism, some children, especially those with average to higher cognitive ability, may only be diagnosed at older ages [6,7]. 

Concerns raised by parents regarding their child’s development have been found to be compatible with a final diagnosis of ASD in the child [8]. When comparing open-ended responses that described concerns raised by parents of toddlers aged 18–24 months who were later diagnosed with ASD to those of parents whose children were not diagnosed (i.e., non-ASD), a notable difference emerged. Though all parents expressed concerns about speech and communication impairments, parents of children later diagnosed with ASD reported more ASD-related concerns, such as social concerns and restricted or repetitive behaviors, including sensory concerns [9]. Sacrey et al. [8] compared the levels of agreement between parents and clinicians regarding early behavioral signs suggestive of autism in high-risk toddlers (i.e., siblings of children diagnosed with ASD) at the ages of 12 and 18 months. Both parents and clinicians filled out a structured questionnaire. Though there was poor agreement between the ratings of parents and professionals during infancy, the day-to-day observations of parents were better able to distinguish between children who would later be diagnosed with ASD (36 months) and those who would not. These findings emphasize the importance of parental reports during the evaluation process of autism. Indeed, the American Academy of Pediatrics recommends that professionals should elicit the concerns of parents, and attend to them, as part of the developmental surveillance process [10]. Despite the accumulated knowledge regarding the importance of parental concerns, there are still studies that report a significant time gap between the age at which parents express their concerns and the age at which the child receives their diagnosis [11].

Parents of children later diagnosed with autism often share their concerns regarding their child’s development with professionals at a very early age, as early as 12 to 24 months [5,12]. Indeed, research on early markers for ASD has been based on parent-reported concerns about specific behaviors presented at a very early age [5,13]. Based on these studies, researchers have described a number of early behavioral precursors related to ASD, including a lack of response to their name, absence of eye contact, lack of nonverbal communication, repetitive movements or posturing of the body, stereotyped or repetitive movement of objects, and more [5,12,14]. 

Based on responses to a single open-ended question, Pfeiffer et al. [15] conducted a qualitative analysis of parental concerns. They categorized parental concerns into twelve subcategories, to compare concerns across different age groups (toddler, preschooler, and those in middle childhood, encompassing ages 1–11 years). Their findings reveal both differences and similarities between these age groups. Notably, all parents expressed concerns within six of the twelve subcategories: ‘atypical behaviors’, ‘sensory’, ‘health’, ‘seeking diagnostic clarity or resources’, ‘developmental’, and ‘motor skills’. Parents of children in the preschool and mid-childhood age groups were more likely to report concerns in the category of behavioral and emotional development, such as children’s ability to express, understand, or regulate their emotions. Conversely, concerns related to the absence of gestures, such as “no pointing”, were more frequently mentioned by parents of children in the toddler and preschool groups. These findings are consistent with those of Sivberg [16], who, through a comprehensive interview with parents, also identified that different behavioral concerns emerged at various stages of childhood.

Following these studies, there has been growing recognition that ASD is characterized by a heterogeneous developmental time course [17]. In some children, typical social communication deficits are already apparent during toddlerhood; in others, the presentation of autism symptoms is gradually manifested [18]. As autistic symptoms and behavioral presentation change over the years, parental concerns regarding their child’s development may also change. However, to date, there has been a paucity of studies examining the types of concerns that are raised by parents of older children in their early and middle childhood years; these concerns may suggest ASD and warrant evaluation. 

The number of referrals for assessment of possible autism has risen dramatically in the past decades. Some referred children exhibit behaviors and social difficulties that are matters of concern, but fail to meet the ASD diagnostic criteria [19,20]. Indeed, one study on the prevalence of final diagnoses of ASD among children (aged 1–19 years) referred to a regional diagnostic autism center revealed that only 61% met ASD diagnosis criteria [21]. Children who were referred for ASD evaluation and did not receive an ASD diagnosis were more frequently diagnosed with attention deficit hyperactivity disorder (ADHD) [21,22,23] and had more co-occurring diagnoses of developmental disorders and medical problems [23]. Other frequent alternative diagnoses included language disorder, disruptive behavior disorder/oppositional defiant disorder, intellectual disability, and anxiety disorder, with most children having multiple diagnoses [21]. Studies that have sought to identify characteristics that may help to distinguish between those who will be diagnosed with ASD and those who will not, have shown that children who receive ASD diagnoses are characterized by lower cognitive ability [23], more severe RRB [22], and poorer abilities in the social communication domain, as determined by professionals [24].

Distinguishing ASD from other disorders can be particularly challenging for clinicians, especially when children are capable of verbal communication and exhibit age-appropriate cognitive abilities. The rationale for this study is twofold: 1. It is essential to distinguish between concerns reported by parents that are linked to specific ASD behaviors, as opposed to behaviors that are not unique to autism and occur in other developmental conditions. This distinction is of paramount importance for primary care physicians and clinicians, as it can facilitate earlier diagnosis and treatment of ASD. 2. Though it is widely recognized that professionals should pay close attention to concerns voiced by parents, previous studies examining parental concerns, as mentioned earlier, have predominantly centered on young children, particularly toddlers [8,9]. Conversely, research focusing on parental concerns for children during their early and middle childhood has been relatively limited.

In the current study, therefore, we focused on children in early and middle childhood within an average cognitive range for whom a professional evaluation of autism was sought. We conducted a study to explore both the prevalence and nature of parental concerns, as well as their concurrence with specific criteria for ASD, as outlined in the DSM-5 [1]. This examination was conducted among parents whose children received a diagnosis of ASD and parents whose children did not receive such a diagnosis. This particular inquiry represents a novel endeavor, as no prior investigations of this nature have been undertaken. To achieve our study goals, we compared two pair-matched groups (age and IQ), with and without a final diagnosis of ASD following a comprehensive ASD evaluation. Two specific study aims were sought, as follows:

1. To compare the frequencies of parental concerns related to each of the seven DSM-5 criteria for ASD (three SCI criteria and four RRB criteria) between pair-matched groups with and without a final diagnosis of ASD.
2. To explore which parental concerns corresponding to specific DSM-5 criteria predict a final diagnosis of ASD.

## 2. Materials and Methods

### 2.1. Participants

The study included 80 participants (57 male and 23 female children), who met the inclusion criteria of being older than 44 months and obtaining an IQ score > 70 on standardized cognitive tests. The age range of the participants was 48–154 months (mean = 98.5, SD = 28.3), and the mean IQ was 92.07 (12.85). The participants were divided into two groups (ASD and non-ASD) based on their final diagnosis following a comprehensive evaluation for suspected autism. The ASD group included 40 participants (8 female children), and the non-ASD group (not meeting diagnostic criteria for ASD) included 40 participants (15 female children). The participants in the two groups were pair-matched by age and cognition (see the Section 3 for participant characteristics). There was no statistical difference in sex distribution across the groups [χ^2^ (1) = 2.99, *p* = 0.08], nor in their demographic characteristics regarding maternal education [F(1,74) = 1.80, *p* = 0.18, µ^2^ = 0.02]. Of interest, in the non-ASD group, final primary diagnoses included mostly ADHD (55%), language disorder (17.5%), and anxiety (17.5%).

### 2.2. Measures

Cognitive Functioning. Cognitive abilities were assessed using one of the following two measures based on the child’s age: the Wechsler Preschool and Primary Scale of Intelligence-Revised [25,26] or the Wechsler Intelligence Scale for Children, 4th Edition [27].

Autism Diagnostic Interview-Revised (ADI-R) [28]. This semi-structured interview is conducted with parents and is designed to diagnose autism in accordance with DSM-IV criteria. To assess the severity of autism, the ADI-R algorithm items related to reciprocal social interactions (RSIs), communication, and restricted/repetitive behaviors (RRBs) are utilized. In the ADI-R scoring system, higher scores are indicative of more severe autism symptoms. This semi-structured interview begins when the examiner asks the parent several open questions. These include: (1) ‘Could you tell me a little about your child?’ (2) ‘Do you have any concerns about the behavior or development of your child in the current period? If so, what are they?’ The parent answers the questions without intermediation or guidance from the examiner.

Autism Diagnostic Observation Scales (ADOS-2) [29]. This semi-structured interactive assessment was designed to evaluate the social and communicative functioning of individuals with ASD. The ADOS total algorithm score was employed to calculate the overall severity score using the ADOS calibrated severity scale (CSS) [30]. The ADOS subdomain scores for social affect (SA) and restricted/repetitive behaviors (RRB) were used to compute the severity score for each subdomain using the calibrated severity scale (CSS) for SA and RRB [31]. Higher ADOS scores indicate more pronounced autism symptomatology in comparison with other children with autism of the same age and language level. A validated translated version of the ADOS was utilized in this study (https://www.wpspublish.com/translations).

Vineland Adaptive Behavior Scales (VABS) [32]. This standardized caregiver interview was developed to evaluate adaptive behaviors in children ranging from birth to 18 years old. It includes four subdomains: communication, daily living skills, socialization, and motor skills, and each of them provides a standard score with a mean (M) of 100 and a standard deviation (SD) of 15. Moreover, it offers an adaptive behavior composite total score with a mean (M) of 100 and standard deviation (SD) of 15. Higher scores indicate more proficient functioning.

Child and Familial Variables. Child characteristics and familial information were collected from the participant’s medical records.

### 2.3. Procedure

This retrospective research study received approval from the Institutional Review Board (Helsinki Committee) of the Medical Center. As the study data were collected retrospectively from medical records and de-identified to protect privacy, the Helsinki Committee did not require parental consent to use the data (Protocol Number: 46-09).

For the entire cohort, data were extracted from the medical records of children referred to the Autism Center between 2015 and 2022. The Autism Center has been engaged in the diagnosis, treatment, and research of autism. All children were referred to the Autism Center for a comprehensive assessment of potential ASD diagnosis and were evaluated by a highly skilled interdisciplinary team. Pediatric developmental neurologists obtained each child’s medical and familial histories and conducted thorough neurological examinations. Cognitive assessments and parental interviews concerning children’s adaptive functioning were conducted by licensed psychologists. ASD was assessed using the Autism Diagnosis Interview-Revised (ADI-R) [28] and Autism Diagnostic Observation Schedule (ADOS-2) [29] by evaluators who achieved the required reliability in administering these tests. The diagnostic criteria for ASD used in the assessment were those outlined in DSM-5 [1].

Parental Concerns Corresponding to Each of the DSM-5 Criteria. Parental concerns were identified from answers given to the introductory question in the ADI-R [28]. In the current study, we analyzed parental responses in reference to the DSM-5 criteria for ASD. These were divided into two domains: (A) Persistent deficits in social communication and social interaction (SCI); (B) Restricted/repetitive behavior (RRB) symptoms. The three criteria of domain A (SCI) are as follows: (1) Deficits in social–emotional reciprocity (A1); (2) Deficits in nonverbal communication (A2); (3) Deficits in developing, maintaining, and understanding relationships (A3). The four criteria of domain B (RRB) are as follows: (1) Stereotyped or repetitive behavior (B1); (2) Excessive adherence to routines (B2); (3) Highly restricted, fixated interests (B3); (4) Hyper- or hyporeactivity to sensory input (B4).

An expert group of two pediatric neurologists and one speech and language pathologist, all with extensive experience in autism diagnosis and treatment, determined which concerns should be categorized under each criterion. Two of the authors reviewed parental responses to the ADI-R introductory question. The content of parental concerns was then analyzed to see whether it corresponded to any of the seven DSM-5 criteria for ASD. If the content of the parental responses corresponded to one of the DSM-5 criteria, the authors coded this criterion as 1; otherwise, the criterion was coded as 0. Parental concerns that did not correspond to any of the DSM-5 criteria (e.g., sleeping problems) were not encoded.

Table 1 gives examples of parental concerns that corresponded to DSM-5 criteria. To verify the reliability of the two authors, 20% of the encodings were reviewed by both authors, and a 90% rate of agreement was found.

### 2.4. Data Analysis

All analyses were performed using SPSS 25. First, the distribution of the variables was examined using skewness and kurtosis tests. All continuous characteristics (age at diagnosis, ADOS-SA-CSS, ADOS-RRB-CSS, ADI-R COMM, ADI-R SI, ADI-R RRB, VABS total score, and IQ) and familial variables (maternal age at birth and maternal education in years) were found to be normally distributed (skewness and kurtosis range = ±1.96).

We compared the ASD and non-ASD groups across several demographic characteristics for both children and parents. Multivariate and one-way analyses of variance (MANOVAs/ANOVAs) were performed for child age at diagnosis, IQ, ADOS-SA-CSS, ADOS-RRB-CSS, ADI-R SI, ADI-R RRB, ADI COMM, VABS total score, maternal age, and maternal years of education. To compare the frequencies of parental concerns that corresponded with each of the seven DSM-5 criteria for ASD (SCI-3 and RRB-4) between the two study groups (ASD; non-ASD), nonparametric chi-squared analyses were performed. A simple logistic regression model was used to assess the predictive value of parental concerns related to each of the seven DSM-5 criteria (independent variable) for inclusion in one of the two groups (ASD, non-ASD) (dependent variables).

## 3. Results

First, we compared the characteristics of the ASD and non-ASD groups. As can be seen in Table 2, the two groups did not significantly differ with respect to age, IQ scores, or VABS composite scores, neither did they differ in terms of familial variables (e.g., maternal education). Both groups had IQ scores within the normal range, and adaptive abilities below the average range. Regarding symptoms of autism, the groups did not differ in the ADI-R subdomain scores that were based on parental reports, but they did significantly differ in their ADOS scores. The ASD group had significantly higher total ADOS-CSS, SA-CSS, and RRB-CSS scores, as assessed by professionals, indicating more prominent autism symptoms.

Next, we compared the percentage of parental concerns that corresponded—based on their content—to each of the seven DSM-5 criteria for ASD, using non-parametric chi-square goodness-of-fit tests (Table 3). Both groups reported significant deficits in developing, maintaining, and understanding social relationships corresponding to the DSM-5 A3 criterion (>80%), excessive adherence to routines corresponding to the DSM-5 B2 criterion (50%), and hyper- or hyporeactivity to sensory input, corresponding to the DSM-5 B4 criterion (30%). The two groups only differed in the frequency of parental reports related to deficits in social–emotional reciprocity (DSM-5 A1 criterion). In the ASD group, parents reported significantly more concerns regarding social–emotional reciprocity than parents in the non-ASD group. Deficits in nonverbal communication were mentioned least by parents in both groups, with only about 12.5% of parents noting such symptoms.

Due to the relatively wide age range within our participant pool, we categorized them into two distinct groups: children in early childhood (ages 4–6) and children in middle childhood (ages 7 and older). In the middle childhood group, which consisted of 25 children with ASD and 24 children without ASD, our analysis yielded results consistent with the overall findings. Specifically, we found significant differences between the two groups only in parental concerns related to social reciprocity (χ^2^ (2, N = 49) = 10.03, *p* = 0.002). In the younger aged group, encompassing 15 children with ASD and 16 children without ASD, we did not observe any significant differences between the two groups in parental concerns related to the DSM criteria for ASD.

Two logistic regressions were performed to ascertain the contribution of parental concerns corresponding to each of the seven DSM-5 criteria for ASD (independent variable) on the final diagnosis (dependent variable). In the first logistic regression, the three criteria relating to impaired social communication/interaction were entered. The logistic regression model was statistically significant, χ^2^ (3, N = 80) = 10.74, *p* = 0.02. The model explained 16.8% (Nagelkerke R^2^) of the variance in the group and correctly classified 67.5% of cases. Only reports on deficits in social–emotional reciprocity (DSM-5 A1 criterion) predicted receipt of an ASD versus a non-ASD diagnosis. Parents in the ASD group were four times as likely to report deficits in social–emotional reciprocity compared with parents in the non-ASD group (OR = 4.76, 95%CI [1.79, 12.68]). In the second logistic regression, the four criteria relating to RRB were entered. In this case, the logistic regression model was not statistically significant, i.e., χ^2^ (3, N = 80) = 0.60, *p* = 0.96.

## 4. Discussion

In the present study, we examined and compared parental concerns in two pair-matched groups of individuals in early and middle childhood, all within the average cognitive range. Children were evaluated to determine the source of their social difficulties. At the end of the process, one group received an ASD diagnosis, and the second group received other non-ASD diagnoses (ADHD, anxiety, or language disorder). We focused on the content of parental concerns raised during the free narrative at the start of the ADI-R interview and examined their relation to the different DSM-5 criteria for ASD.

Considering the seven DSM-5 criteria for ASD, only the parental concerns corresponding to the DSM-5 criterion ‘Deficits in social–emotional reciprocity’ (A1) served to distinguish between the two groups (ASD vs. non-ASD), as parents of children diagnosed with ASD reported significantly more concerns related to this criterion. Evaluating the significance of parental concern regarding reciprocity revealed that this was a high predictor of a final ASD diagnosis.

It appears that initial concerns regarding difficulties in social relationship are most likely to lead parents to seek evaluation for autism. Of the seven DSM-5 criteria for ASD, reports on ‘deficits in developing, maintaining and understanding relationships’ (DSM-5 A3 criterion) were the most frequent parental concern in both groups. In addition, about half of the parents reported difficulties with changes in routines (DSM-5 B2 criterion), a third reported sensory modulation problems (DSM-5 B4 criterion), and about 20–25% reported ‘highly restricted, fixated interests’ (DSM-5 B3 criterion) or ‘stereotyped or repetitive behavior’ (DSM-5 B1 criterion). It is noteworthy that concerns regarding ‘deficits in nonverbal communication’ (DSM-5 A2 criterion) were hardly mentioned by parents.

In the current study, most parents in both groups raised concerns regarding their child’s social difficulties. Children aged 4–13 years are expected to develop and establish social relationships [33,34]. It is reasonable to assume that the absence of these significant relationships, particularly in older children with average cognitive ability, would be very noticeable for parents and elicit great concern. Children in our study who were not diagnosed with ASD were diagnosed with language disorder, ADHD, or anxiety, with most of them receiving a diagnosis for ADHD. It is known that these two diagnoses (ASD and ADHD) have overlapping symptoms, and both impact social relationships [35,36,37]; thus, it is not surprising that parents of both groups frequently report on social difficulties. In contrast, concerns regarding ‘deficits in nonverbal communication’ were least frequently raised by parents from both groups. This finding is somewhat surprising as, at younger ages (toddlerhood), absence of eye contact or lack of nonverbal communication is often raised by parents as a major concern [16]. These symptoms are considered early precursors to an ASD diagnosis [5,12,14]. One possible explanation is that participants in our study were verbally fluent, had average to normal cognitive ability, and probably used verbal means to communicate with their partners. Thus, the absence of nonverbal communication may be less noticeable for parents of verbally fluent children compared with parents of nonverbal toddlers, who are supposed to use gestures and eye contact to communicate needs or share interests and joy. These findings align with previous findings [15,16] that concerns raised by parents vary depending on their child’s developmental stage. Concerns regarding behaviors at younger ages may be less pronounced in older ages, considering the child’s abilities and behaviors.

Parental concerns regarding ‘excessive adherence to routines’ were raised by approximately half of the parents in each group. Approximately one-third of parents raised concerns regarding ‘hyper-or hyporeactivity to sensory input’, whereas concerns regarding ‘stereotyped or repetitive behavior’ and ‘highly restricted, fixated interests’ were raised by approximately a quarter of parents in the present study. These findings were surprising, as these criteria are all highly associated with autism. It is possible that in the non-ASD group, children diagnosed with anxiety tended to be more rigid and used compulsive behavior to alleviate their anxiety levels. Additionally, children diagnosed with ADHD and anxiety in the non-ASD group may have experienced greater difficulties in sensory processing modulation, so that differences between the groups were not significant. Overall, such behaviors can impact the quality of family life, regardless of ASD status, and are therefore likely to be noticed by parents and reported to professionals.

The most important finding of the current study is that only the parental concerns corresponding to the DSM-5 ASD criterion ‘deficits in social–emotional reciprocity’ served to significantly distinguish between the ASD and non-ASD groups. Parents of children with a final diagnosis of ASD more frequently identified behaviors that characterized a lack of social reciprocity (e.g., difficulties engaging in small talk). A lack of social–emotional reciprocity refers to an abnormal social approach, with failure to maintain normal back-and-forth conversation, as well as reduced sharing of interests, emotions, or affect, and/or failure to initiate or respond to social interactions [1]. Engaging in social–emotional reciprocity begins to develop at very young ages (when an infant smiles back at a parent or responds when he hears his name) and continues throughout life (when the child shares events with the parent). These reciprocal social abilities develop gradually and form the foundations for interpersonal relationships. Difficulties in social–emotional reciprocity appear to be more unique to autism than the other criteria in DSM-5, which can characterize other pathologies (i.e., deficits in sensory modulation or difficulties in developing social relationships). Therefore, based on these findings, professionals should be alarmed and carefully consider parental concerns regarding social–emotional reciprocity.

Furthermore, this study delved into parental concerns by analyzing more homogenous age groups, specifically, children in early childhood and middle childhood were examined separately. Notably, significant differences in parental concerns, similar to the findings for the whole group, were observed only within the middle-childhood group. It is plausible that the smaller sample size in the early childhood group may have influenced this outcome, underscoring the need for future investigations using a larger cohort.

Although it was not the focus of our study, it is essential to address the discrepancy between ADI-R and ADOS scores for the assessment of autistic symptoms. Though the two groups in our study did not differ with respect to their ADI-R subdomain scores, they did significantly differ in their ADOS scores. The ADI-R and ADOS are two diagnostic tools for ASD that are noted for their high sensitivity and specificity and are thus considered ‘gold standard’ diagnostic tools for ASD diagnosis [38]. Grzadzinski et al. [24] compared autistic symptoms as expressed during the ADOS and ADI-R in children referred to ASD specialty clinics who received diagnoses of ADHD or ASD. In line with the findings of the present study, they found that ADOS scores in both domains (SA and RRB), assessed by professionals, were better at distinguishing between the two diagnostic groups. In addition, a recent systematic review and meta-analysis indicated that the ADOS-2 is more accurate than ADI-R in clinical settings [39]. With the increasing prevalence of autism, and greater public awareness of the condition, parents may tend to suspect autism and seek a full evaluation when their children experience social difficulties and problems forming adequate friendships [24].

The current study has an important clinical implication. When parents seek professional evaluation to assess their child’s social difficulties, primary care providers should be specifically aware of and attentive to parental concerns regarding social–emotional reciprocity, particularly when it comes to school-age children with normal cognitive ability. In light of the findings from the current study, clinicians are encouraged to inquire specifically about fundamental early social reciprocity behaviors that could potentially signal a heightened suspicion for autism. These behaviors include deficits in joint attention, sharing of interests and enjoyment, unresponsiveness when addressed by name, and a lack of reciprocal enjoyment. When such parental concerns are raised in community clinics in addition to concerns related to other ASD behaviors, clinicians should refer the child immediately for a more extensive evaluation of ASD. Furthermore, given the inherent role of parents as the primary caregivers for their children, it is imperative to acknowledge the variability in parental experience levels. Consequently, it becomes essential to furnish parents with initial guidance aimed at discerning potential indicators of atypical development in their child, particularly within the domain of social development. Additionally, it is of paramount importance to offer recommendations regarding appropriate channels to seek assistance and identifying suitable contacts in the event of any concerns.

The current study has several strengths; these include the use of pair-matched groups (ASD and non-ASD) for age and cognitive ability, as this methodology minimized the diversity between the two groups. Additionally, all children underwent extensive evaluation of ASD and other comorbidities using standardized tests or questionnaires.

### Limitations

The decision regarding the correspondence between the content of parental concerns and relevant DSM-5 criteria for autism diagnosis was based on agreement between the expert group, not on a standardized tool.

## 5. Conclusions

Our investigation aimed to determine which parental concerns corresponding to specific DSM-5 criteria could predict an ultimate diagnosis of ASD. Our results pertaining to these objectives revealed a strong and predictive link between parental concerns related to challenges in social–emotional reciprocity and a final diagnosis of ASD. Notably, this particular concern appeared to be distinctive to ASD and was less frequently cited for children receiving alternative diagnoses.

### Future Studies

We suggest that future studies examine parental concerns for children in the same age range, but with lower IQs. Additionally, it is essential that research is extended to compare concerns raised by parents with respect to sex differences (male vs. female). In the current study, the groups did not differ in terms of their familial characteristics (maternal education); concerns raised by more diverse ethnic and cultural populations should be addressed in future research. Moreover, it is prudent to replicate this research using a more extensive age range encompassing younger children, particularly those possessing cognitive abilities within a normal range.

## Figures and Tables

**Table 1:** Examples of parental concerns that corresponded to DSM-5 criteria.

Domain		Criterion	Examples of Parental Concerns
Domain A: persistent deficits in social communication and social interaction across multiple contexts.	A1	Deficits in social–emotional reciprocity.	Failure to respond to one’s name; lives in his own world; rarely shares interests or achievements with parents; difficulties in back-and-forth conversation or small talk; overly friendly and comfortable with strangers; unshared laughter.
	A2	Deficits in nonverbal communication.	Abnormalities in eye contact; lack of facial expressions; lack of pointing gestures.
	A3	Deficits in developing, maintaining, and understanding relationships.	Reduced interest in peers; difficulties in playing with peers; plays by himself or tends to play with younger children; difficulties with shared imaginative play; does not remember his peers’ names.
Domain B: restricted/repetitive behavior (RRB) symptoms.	B1	Stereotyped or repetitive behaviors.	Lining up toys or flipping objects; repetitive and unusual body movements or noises; opening and closing drawers or doors; asking about the same thing repeatedly.
	B2	Insistence on sameness, inflexible adherence to routines, or ritualized patterns.	Extreme distress at small changes; difficulty with transitions; rigid thinking patterns; the need for the same route or food every day.
	B3	Highly restricted, fixated interests.	Excessive interest in cars, wheels, computer games, letters, numbers, and Bible reading.
	B4	Hyper- or hyporeactivity to sensory input.	Adverse response to sounds; refusing to wear certain clothes; avoids getting ‘messy’; dislikes food of particular texture; avoids kisses and hugs; excessively touching objects.

**Table 2:** Participant characteristics.

Variables	ASD Mean (SD) *n* = 40	Non-ASD Mean (SD) *n* = 40	F	µ^2^
Age in months IQ	99.70(29.14) 90.25(12.89)	97.3(24.89) 93.75 (13.37)	0.16 1.36	0.002 0.02
VABS	71.95(10.30)	74.07(8.83)	0.97	0.01
Autism severity				
ADOS-CSS	8.80(3.11)	3.27(2.56)	99.77 ***	0.56
ADOS-SA-CSS	8.15(1.61)	3.57(2.77)	79.93 ***	0.51
ADOS-RRB-CSS	7.26(2.40)	4.55(2.80)	25.40 ***	0.26
ADI-R				
ADI-RSI	13.95(5.75)	11.95(4.74)		
ADI-R Communication	11.54(5.60)	10.29(4.61)	0.97	0.04
ADI-R RRB	6.13(2.57)	5.34(2.67)		

*** *p* < 0.001. VABS = Vineland Adaptive Behavior Scale; ADOS-CSS = Autism Diagnostic Observation Scales—Calibrated Severity Scales; ADOS-SA-CSS = Autism Diagnostic Observation Scales—Social Affect—Calibrated Severity Scales; ADOS-RRB-CSS = Autism Diagnostic Observation Scales—Restricted and Repetitive Behavior—Calibrated Severity Scales; ADI-R RSI = Autism Diagnostic Interview-Revised—Reciprocal Social Interactions; ADI-R RRB = Autism Diagnostic Interview-Revised—Restricted and Repetitive Behaviors.

**Table 3:** Frequency of the criteria from DSM-5 domains for ASD in parental concerns.

	ASD (*n* = 40)	Non-ASD (*n* = 40)	χ^2^	*p*
Deficits in social–emotional reciprocity (A1)	75% (30)	40% (16)	10.03 *	0.002
Deficits in nonverbal communication (A2)	12.5% (5)	12.5% (5)	0.00	1.0
Deficits in developing, maintaining, and understanding social relationships (A3)	87.5% (35)	85% (34)	0.105	0.75
Stereotyped or repetitive behavior (B1)	25% (10)	25% (10)	0.00	1.0
Excessive adherence to routines (B2)	55% (22)	47.6% (20)	0.20	0.65
Highly restricted, fixated interests (B3)	22.5% (9)	20% (8)	0.78	0.75
Hyper- or hyporeactivity to sensory input (B4)	32.5% (13)	37.5% (15)	0.22	0.64

* *p* < 0.5.

## Data Availability

The data presented in this study are available on request from the corresponding author.

## References

[1] American Psychiatric Association (2013). Diagnostic and Statistical Manual of Mental Disorders (DSM-5®).

[2] Hyman S.L., Levy S.E., Myers S.M. (2020). Identification, Evaluation, and Management of Children with Autism Spectrum Disorder. Pediatrics.

[3] Ben-Itzchak E., Zachor D.A. (2020). Toddlers to teenagers: Long-term follow-up study of outcomes in autism spectrum disorder. Autism.

[4] Clark M.L.E., Vinen Z., Barbaro J., Dissanayake C. (2017). School age outcomes of children diagnosed early and later with autism spectrum disorder. J. Autism Dev. Disord..

[5] Zwaigenbaum L., Bauman M.L., Choueiri R., Kasari C., Carter A., Granpeesheh D., Mailloux Z., Smith Roley S., Wagner S., Fein D. (2015). Early intervention for children with autism spectrum disorder under 3 years of age: Recommendations for practice and research. Pediatrics.

[6] Saban-Bezalel R., Zachor D.A., Ben-Itzchak E. (2022). Relationship between cognitive ability and predictors for age at the time of autism spectrum disorder diagnosis. Psychiatry Res..

[7] Zwaigenbaum L., Duku E., Fombonne E., Szatmari P., Smith I.M., Bryson S.E., Bruno R. (2019). Developmental functioning and symptom severity influence age of diagnosis in Canadian preschool children with autism. Paediatr. Child. Health.

[8] Sacrey L.A.R., Zwaigenbaum L., Bryson S., Brian J., Smith I.M., Roberts W., Garon N. (2018). Parent and clinician agreement regarding early behavioral signs in 12-and 18-month-old infants at-risk of autism spectrum disorder. Autism Res..

[9] Richards M., Mossey J., Robins D.L. (2016). Parents’ concerns as they relate to their child’s development and later diagnosis of autism spectrum disorder. J. Dev. Behav. Pediatr..

[10] Lipkin P.H., Macias M.M., Norwood K.W., Brei T.J., Davidson L.F., Davis B.E., Voigt R.G. (2020). Promoting optimal development: Identifying infants and young children with developmental disorders through developmental surveillance and screening. Pediatrics.

[11] Zuckerman K.E., Lindly O.J., Sinche B.K. (2015). Parental concerns, provider response, and timeliness of autism spectrum disorder diagnosis. J. Pediatr..

[12] Wetherby A.M., Woods J., Allen L., Cleary J., Dickinson H., Lord C. (2004). Early indicators of autism spectrum disorders in the second year of life. J. Autism Dev. Disord..

[13] Yirmiya N., Charman T. (2010). The prodrome of autism: Early behavioral and biological signs, regression, peri-and post-natal development and genetics. J. Child. Psychol. Psychiatry.

[14] Guinchat V., Chamak B., Bonniau B., Bodeau N., Perisse D., Cohen D., Danion A. (2012). Very early signs of autism reported by parents include many concerns not specific to autism criteria. Res. Autism Spectr. Disord..

[15] Pfeiffer D., Holingue C., Dillon E., Kalb L., Reetzke R., Landa R. (2021). Parental concerns of children with ASD by age: A qualitative analysis. Res. Autism Spectr. Disord..

[16] Sivberg B. (2003). Parents’ detection of early signs in their children having an autistic spectrum disorder. J. Pediatr. Nurs..

[17] Lord C., Brugha T.S., Charman T., Cusack J., Dumas G., Frazier T., Jones E.J.H., Jones R.M., Pickles A., State M.W. (2020). Autism spectrum disorder (primer). Nat. Rev. Dis. Primers.

[18] Bolton P.F., Golding J., Emond A., Steer C.D. (2012). Autism spectrum disorder and autistic traits in the Avon Longitudinal Study of Parents and Children: Precursors and early signs. J. Am. Acad. Child. Adolesc. Psychiatry.

[19] Constantino J.N., Todd R.D. (2003). Autistic traits in the general population: A twin study. Arch. Gen. Psychiatry.

[20] Robinson E.B., Munir K., Munafo M.R., Hughes M., McCormick M.C., Koenen K.C. (2011). Stability of autistic traits in the general population: Further evidence for a continuum of impairment. J. Am. Acad. Child. Adolesc. Psychiatry.

[21] Monteiro S.A., Spinks-Franklin A., Treadwell-Deering D., Berry L., Sellers-Vinson S., Smith E., Voigt R.G. (2015). Prevalence of autism spectrum disorder in children referred for diagnostic autism evaluation. Clin. Pediatr..

[22] Avlund S.H., Thomsen P.H., Schendel D., Jrgensen M., Clausen L. (2021). Time Trends in Diagnostics and Clinical Features of Young Children Referred on Suspicion of Autism: A Population-Based Clinical Cohort Study, 2000–2010. J. Autism Dev. Disord..

[23] Smith I.C., Swain D., Murphy H.G., Ollendick T.H., White S.W. (2019). The Under- and Over-Identification of Autism: Factors Associated with Diagnostic Referral. J. Clin. Child Adolesc. Psychol..

[24] Grzadzinski R., Dick C., Lord C., Bishop S. (2016). Parent reported and clinician-observed autism spectrum disorder (ASD) symptoms in children with attention defcit/hyperactivity disorder (ADHD): Implications for practice under DSM-5. Mol. Autism.

[25] Wechsler D. (1989). Manual for the Wechsler Preschool and Primary Scale of Intelligence—Revised (WPPSI-R).

[26] Lieblich A., Shinar M. (1975). The predictive validity of the WPPSI with Israeli children. Educ. Psychol. Meas..

[27] Wechsler D. (2010). WISC-IV HEB. Wechsler Intelligence Scale for Children.

[28] Lord C., Rutter M., Le Couteur A. (1994). Autism diagnostic interview-revised: A revised version of a diagnostic interview for caregivers of individuals with possible pervasive developmental disorders. J. Autism Dev. Disord..

[29] Lord C., Rutter M., DiLavore P.C., Risi S., Gotham K., Bishop S. (2012). Autism Diagnostic Observation Schedule.

[30] Gotham K., Pickles A., Lord C. (2009). Standardizing ADOS scores for a measure of severity in autism spectrum disorders. J. Autism Dev. Disord..

[31] Hus V., Gotham K., Lord C. (2014). Standardizing ADOS domain scores: Separating severity of social affect and restricted and repetitive behaviors. J. Autism Dev. Disord..

[32] Sparrow S.S., Cicchetti D.V., Balla D.A. (2005). Vineland Adaptive Behavior Scales.

[33] Eccles J.S. (1999). The development of children ages 6 to 14. Future Child..

[34] Hartup W.W. (1989). Social relationships and their developmental significance. Am. Psychol..

[35] Craig F., Lamanna A.L., Margari E., Simone M., Margari L. (2015). Overlap between Autism Spectrum Disorders and Attention Deficit Hyperactivity Disorder Searching for Distinctive/Common Clinical Features. Autism Res..

[36] De Boo G.M., Prins P.J. (2007). Social incompetence in children with ADHD: Possible moderators and mediators in social-skills training. Clin. Psychol. Rev..

[37] Ragnarsdóttir B., Hannesdóttir D.K., Halldorsson F., Njardvik U. (2018). Gender and Age Differences in Social Skills among Children with ADHD: Peer Problems and Prosocial Behavior. Child. Fam. Behav. Ther..

[38] Falkmer T., Anderson K., Falkmer M., Horlin C. (2013). Diagnostic procedures in autism spectrum disorders: A systematic literature review. Eur. Child. Adolesc. Psychiatry.

[39] Lebersfeld J.B., Swanson M., Clesi C.D., O’Kelley S.E. (2021). Systematic review and meta-analysis of the clinical utility of the ADOS-2 and the ADI-R in diagnosing autism spectrum disorders in children. J. Autism Dev. Disord..

